# SecMet-FISH: labeling, visualization, and enumeration of secondary metabolite producing microorganisms

**DOI:** 10.1093/femsec/fiae038

**Published:** 2024-03-15

**Authors:** Yannick Buijs, Aileen Ute Geers, Iuliana Nita, Mikael Lenz Strube, Mikkel Bentzon-Tilia

**Affiliations:** Department of Biotechnology and Biomedicine, Technical University of Denmark, 2800 Kongens Lyngby, Denmark; Department of Biotechnology and Biomedicine, Technical University of Denmark, 2800 Kongens Lyngby, Denmark; Department of Biotechnology and Biomedicine, Technical University of Denmark, 2800 Kongens Lyngby, Denmark; Department of Biotechnology and Biomedicine, Technical University of Denmark, 2800 Kongens Lyngby, Denmark; Department of Biotechnology and Biomedicine, Technical University of Denmark, 2800 Kongens Lyngby, Denmark

**Keywords:** biosynthetic gene clusters, fluorescence in situ hybridization, functional genes, geneFISH, in-solution FISH, secondary metabolites

## Abstract

Our understanding of the role of secondary metabolites in microbial communities is challenged by intrinsic limitations of culturing bacteria under laboratory conditions and hence cultivation independent approaches are needed. Here, we present a protocol termed Secondary Metabolite FISH (SecMet-FISH), combining advantages of gene-targeted fluorescence *in situ* hybridization (geneFISH) with in-solution methods (in-solution FISH) to detect and quantify cells based on their genetic capacity to produce secondary metabolites. The approach capitalizes on the conserved nature of biosynthetic gene clusters (BGCs) encoding adenylation (AD) and ketosynthase (KS) domains, and thus selectively targets the genetic basis of non-ribosomal peptide and polyketide biosynthesis. The concept relies on the generation of amplicon pools using degenerate primers broadly targeting AD and KS domains followed by fluorescent labeling, detection, and quantification. Initially, we obtained AD and KS amplicons from *Pseuodoalteromonas rubra*, which allowed us to successfully label and visualize BGCs within *P. rubra* cells, demonstrating the feasibility of SecMet-FISH. Next, we adapted the protocol and optimized it for hybridization in both Gram-negative and Gram-positive bacterial cell suspensions, enabling high-throughput single cell analysis by flow cytometry. Ultimately, we used SecMet-FISH to successfully distinguish secondary metabolite producers from non-producers in a five-member synthetic community.

## Teaser

SecMet-FISH is a novel protocol enabling detection and quantification of microbial cells based on their genetic capacity for secondary metabolite production.

## Introduction

The significance of chemically mediated interactions among microorganisms is well established, yet the importance of the diversity of microbial secondary metabolites in shaping the assembly and dynamics of microbial communities remains poorly understood. This is in large part due to the intrinsic limitations of culturing environmental bacteria under laboratory conditions, which result in a significant gap between the number of bacterial lineages we can culture and analyze under manmade conditions, and the number we can detect in nature by culture independent methods (Steen et al. 2019). Moreover, complexity and spatial organization is crucial for understanding how secondary metabolite producers are distributed and how they influence microbial community assembly, composition, and function; elements which are excluded in the analyses of laboratory monocultures.

Metagenomic sequencing of environmental samples has facilitated culture independent discovery of novel taxa and functional genes (Rinke et al. 2013). However, such approaches also omit spatial organization and microscale variability. Moreover, this approach has the inherent disadvantage of sequencing all DNA, be it informative or not, which means that sequences of interest are diluted within data of secondary interest, especially in highly complex natural microbial communities (Zaheer et al. 2018, Libis et al. 2019, Robinson et al. 2021). While metagenomic assembly and binning methods have improved dramatically in the last few years (Wu et al. 2016, Nurk et al. 2017, Nissen et al. 2021), tying functional genes to specific taxa remains an additional challenge when relevant phylogenetic markers and functional genes of interest are distributed across contigs in incomplete assemblies. Moreover, genetic regions with high levels of repetition and tandem repeats (such as in biosynthetic gene clusters, or BGCs) are notorious breakpoints in genome assemblies, as exemplified by an extreme level of fragmentation of metagenomically-derived BGCs. By contrast, development of fluorescence *in situ* hybridization (FISH) based approaches using polynucleotide probes has the advantage of direct visualization rather than computational inference through the labelling of bacterial cells based on the presence or absence of specific genes (Moraru et al. 2010, Barrero-Canosa et al. 2017). This technique is routinely used to couple bacterial phylogeny to functionality and investigate where these cells are localized *in situ* (Ansorge et al. 2019, Richards and Mattes 2021). However, current approaches are challenged with limited signal intensity of single-copy target sequences and the fact that cells are first chemically fixed and immobilized on a surface, which prohibits selective extraction and downstream analysis. To circumvent this, conventional FISH methods e.g. targeting bacterial ribosomes have been adapted for ‘in-solution’ use, enabling high throughput analyses of cell populations by flow cytometry (Flow-FISH; Yilmaz et al. 2010, Haroon et al. 2013, Freen-van Heeren 2021) as well as enrichment of targeted phylogenetic groups by fluorescence-activated cell sorting (FACS; Podar et al. 2007). Combined with metagenomics of FACS-enriched bacterial communities, or single cell sequencing, Flow-FISH has provided genomic sequences of rare and elusive community members (Podar et al. 2007, Grieb et al. 2020). These methods have, however, focused on selection of microorganisms based on their phylogeny, or gene expression, rather than their genetic potential to produce specific metabolites.

Combining the potential of direct-geneFISH with that of in-solution FISH methodology, we aimed to establish an approach that allows for fluorescent labeling, visualization and in-solution enumeration of microorganisms based on their biosynthetic potential. We envisioned that detection, quantification, and potentially fluorescence-guided extraction (e.g. FACS or optical trapping) of bacteria rich in biosynthetic genes would be a valuable tool for understanding the role of secondary metabolite producing microorganisms in the environment, but also for genome mining and drug discovery (Robinson et al. 2021, Geers et al. 2022), as secondary metabolites play a significant role in modern medicine, serving as the inspiration for small-molecule therapeutics. To accomplish this, we aimed to leverage the conserved and modular nature of the adenylation (AD) and ketosynthase (KS) domains, both involved in the biosynthesis of secondary metabolites, including compounds with e.g. antibiotic activities (Walsh 2016). These gene domains are readily amplified with PCR through the use of established degenerate primers (Piel 2002, Ayuso-Sacido and Genilloud 2005, Geers et al. 2022), and their modular organization ensures a series of target sequences for hybridization, allowing for signal intensities sufficient for detection across instrumentation with different sensitivities. First, we set out to show that a mix of PCR amplicon sequences, rather than one specific sequence, can successfully be used to synthesize polynucleotide probes and label target cells. Next, we aimed to adapt the protocol for labelling of Gram-positive and Gram-negative bacterial cells in suspension to enable high-throughput detection and quantification using flow cytometry. Lastly, we assessed whether the fluorescence signal intensities generated by the SecMet-FISH approach are sufficient for selective identification of secondary metabolite producers in a synthetic community.

## Materials and methods

### Strains and growth conditions

SecMet-FISH was developed and optimized using the cultured strains *Pseodoalteromonas rubra* S4059 (Vynne et al. 2011) and the *Streptomyces coelicolor* M145 (lab maintained *S. coelicolor* A3 (Bentley et al. 2002) strain with accumulated mutations and plasmid deletions, for genomic sequence see NCBI accession GCA_008931305.1; Tong et al. 2015). In a synthetic community experiment (see “Synthetic Community Enrichment Experiment”, below), the strains *Pseudoalteromonas mariniglutinosa* DSM15203 (Romanenko et al. 2003), *Aliivibrio fischeri* MJ11 (Urbanczyk et al. 2007, Mandel et al. 2009), *Vibrio anguillarum* NB10 (Holm et al. 2015) and *Escherichia coli* MG1655 (Zhang et al. 2019) were used. *Pseudoalteromonas rubra, P. mariniglutinosa, A. fischeri* and *V. anguillarum* cells were cultured in 20 ml Marine Broth (BD Biosciences) at 25°C with 200 r/m orbital shaking for 24 h. *Streptomyces coelicolor* cells were cultured in ISP2 broth, consisting of 4 g yeast extract, 10 g malt extract and 4 g glucose in 1 L tap H_2_O at 30°C with 200 r/m orbital shaking for 48 h. *Escherichia coli* cells were cultured in LB broth (BD Biosciences) at 37°C with 200 r/m orbital shaking for 24 h. Cultures of *P. rubra, P. mariniglutinosa, A. fischeri, V. anguillarum* and *E. coli* were re-inoculated and grown to an OD600 of 0.3–0.8 (approximately 4 h of growth), and *S. coelicolor* cultures were grown for 48 h after inoculation from a spore cryostock (OD600 = 1.0–2.0).

#### DNA extractions and PCR amplification of biosynthetic domains

DNA from *P. rubra, S. coelicolor* and the synthetic community (SynCom) samples were extracted using the NucleoSpin Tissue kit (740952.250 Machery-Nagel, Germany). PCRs were performed for two applications: 1) to amplify the conserved DNA sequences encoding the AD and KS domains, as well as the Non-Poly 350 sequence, for downstream fluorophore attachment to synthesize SecMet-FISH probes (see below), and 2) for amplicon sequencing of the conserved biosynthetic AD and KS domains. All PCR amplifications were synthesized with HotStarTaq polymerase (Qiagen) in 50 µL reaction volumes. Detailed information on primer sequences, thermal cycling conditions, and reagent and template concentrations are summarized in Table S1.

Amplification of the AD and KS domains from *P. rubra* and *S. coelicolor* gDNA was achieved directly in a one-step PCR, except for the AD amplification from *P. rubra*. Due to an additional, putative unspecific product (as checked by agarose gel analysis), a two-step PCR was employed for AD amplification from *P. rubra*. PCR product of the first round (38 cycles) was separated on an 1.5% agarose gel after which the ≈700 bp band was excised and gel purified using the GFX PCR DNA purification kit (Illustra, USA) and eluted in 25 µL elution buffer (10 mM Tris, 1 mM EDTA). Gel-purified products were diluted to a concentration of 1–10 pg/µL and used as template for the second PCR round consisting of 35 cycles. For amplicon sequencing purposes, 8 bp-indexed primers were used in this second round PCR.

#### Amplicon sequencing and analysis

Amplicons of the biosynthetic AD and KS domains were sequenced by Novogene (Cambridge, UK) on an Illumina Novaseq 6000. All fastq files were demultiplexed using cutadapt v1.18 (Martin 2011) and quality filtered with fastp v0.20 (Chen et al. 2018), using default settings. Briefly, sequence pairs with more than 40% bases <15 Phred-score were removed, and all sequences were 3′ trimmed by quality, i.e. bases in a sliding window having a mean Phred-score <15 were trimmed.

For analysis of the AD and KS domains amplified from the model strain *P. rubra*, demultiplexed reads were mapped to the reference genome (NCBI accession: GCA_005886805.2 and GCA_008931305.1) with bowtie2 v2.3.5 (Langmead and Salzberg 2012) using default options except for the maximum fragment length which was set to 1000 bp. Mapped regions were analyzed using the mpileup function of samtools 1.6 (Li et al. 2009) and further processed and visualized with custom R scripts. In order to quantify the level of *in vitro* PCR-amplification and sequencing of AD and KS domains from *P. rubra*, a custom database of BGCs and their locations in this genome was built using antiSMASH v6.0 (Blin et al. 2021). The genomic coordinates of these BGCs were then cross-referenced with the bowtie2 mappings, and domains were considered successfully amplified if the number of mapped reads at their genomic location amounted to more than 1% of the total mapped reads.

#### Polynucleotide probe synthesis for SecMet-FISH

Gene probes were synthesized by chemical labelling with the Alexa Fluor 488, 594 and 647 fluorophores using the Ulysis Nucleic Acid Labeling kit (Life Technologies, USA). First, AD and KS amplicons from the same target sample were pooled and cleaned up with the AMPure XP magnetic beads with an elution volume of 25 µL in labelling buffer from the labeling kit. Subsequently, 1 µg of pooled amplicon DNA was labelled following the protocol described direct-geneFISH (Barrero-Canosa et al. 2017). For each fluorophore, a non-sense control polynucleotide probe NonPolyPr350 (Moraru et al. 2010) was synthesized using the same procedures, and used as a negative control probe in all SecMet-FISH experiments.

#### SecMet-FISH on immobilized cells

Prior to the *in situ* hybridization reactions, cells were fixed in 1% (v/v, final concentration) paraformaldehyde for one hour at room temperature. Fixed cells were pelleted by centrifugation for 2 min at 10 000 × *g*, washed twice in PBS (8.0 g/L NaCl, 0.2 g/L KCl, 1.15 g/L Na_2_HPO_4_, 0.2 g/L NaH_2_PO_4_, pH = 7.3) and stored in 50% PBS-EtOH at −20°C. For SecMet-FISH on surface-immobilized cells, approximately 10^7^ fixed cells were vacuum filtered onto a 0.2 µm (pore size) membrane filter (Whatmann, diameter = 45 mm). The membrane filter was cut into triangular pieces of approximately 1 cm^2^, which were used for hybridization according to (Barrero-Canosa et al. 2017) using a probe concentration of 100 pg/µL in a hybridization buffer consisting of 40% (v/v) formamide, 900 mM NaCl, 20 mM Tris-HCl pH = 7.4, 0.1 mg/mL DNA from salmon sperm, 0.01% (w/v) SDS, and 10% (w/v) dextran sulfate.

#### SecMet-FISH in solution

To start the in-solution SecMet-FISH protocol targeting Gram-negative bacteria, fixed cells were pelleted by centrifugation in PCR tubes for 2 min at 10 000 × *g* and washed twice with PBS. Cells were resuspended in 50 µL hybridization buffer consisting of 40% (v/v) formamide, 900 mM NaCl, 20 mM Tris-HCl pH = 7.4, 0.1 mg/mL DNA from salmon sperm, 0.01% (w/v) SDS and 10% (w/v) dextran sulfate and sonicated for 1–2 min to aid resuspending the cell pellet. Polynucleotide probes were added to a final concentration of 250 pg/µL for hybridization. Double stranded target DNA and probe DNA were denatured for 20 min at 80°C and hybridization reactions were incubated for 16 h at 46°C. Following hybridization, cells were pelleted and washed with pre-warmed 48°C washing buffer (46 mM NaCl, 20 mM Tris-HCl pH 7.4, 5 mM EDTA pH 8.0 and 0.01% (w/v) SDS), pelleted again and incubated at 48°C for 20 min in pre-warmed washing buffer. Finally, cells were centrifuged, washed one time with PBS and resuspended in 50–100 µL of PBS.

For the adjustment of the protocol to target the Gram-positive *S. coelicolor*, the following modifications were made. PBS-washed cells were treated with 0.5 mg/mL lysozyme in 0.1 M Tris-HCl pH 7.4 for 30 min at 37°C and washed twice with PBS. Lysozyme treated cell suspensions were not subjected to sonication for resuspension of the pellet. Denaturation was performed at 75°C for 20 min, and the hybridization buffer contained 50% (v/v) formamide, whereas the washing buffer NaCl concentration was adjusted to 18 mM.

#### Microscopy analysis

Hybridized cell suspensions were stained with 2 µg/mL (final concentration) DAPI, incubated for 10 min in the dark, after which 3 µL of cell suspension was pipetted on top of a coverslip, mixed with 3 µL Vectashield mounting medium and covered by a 5×5×1 (length x width x height) mm agarose (1% w/v in milliQ water) pad. Mounted samples were observed with an inverted epifluorescence Nikon Ti2 microscope equipped with a Prime BSI Scientific CMOS (Teledyne Photometrics) camera, using a 60 × oil immersion plan apochromatic objective. Alexa488 probe signal was recorded with a 470 nm lamp and the GFP-3035D filterset using an exposure time of 200 ms; Alexa594 probe signal was recorded with a 555 nm lamp and the mCherry-C filterset using an exposure time of 200 ms; DAPI signal was recorded with a 395 nm lamp and the DAPI-5060C filterset using an exposure time of 50 ms. Sets of images of the same sample type, including the non-sense negative control probe labelled with Alexa594, were processed in ImageJ using the same procedure and settings.

To quantify the degree of association between DAPI and Alexa594 probe signal in in-solution SecMet-FISH, images of triplicate *P. rubra* samples labelled with DAPI and the Alexa594-labelled SecMet probes were analyzed in ImageJ, using the ‘Count cells' function. Here, all instances of DAPI and/or Alexa594 signal was summarized in a 2×2 contingency table according to co-occurrence.

#### Flow cytometry analysis

Flow cytometry was employed for analysis of SecMet-FISH in solution on a MACSQuant VYB flow cytometer (Miltenyi Biotec, Germany). Cytograms were recorded using SSC as trigger and cells were discriminated from noise by gating DAPI-stained cells in the V1 channel with a 405 nm laser and 450/50 nm filter. SecMet-FISH signals were recorded in the B1 channel with a 488 nm laser and 525/50 nm filter for Alexa488 labelled probes, the Y2 channel with a 561 nm laser and 615/20 nm filter for Alexa594 labelled probes and the Y3 channel with a 561 nm laser and 661/20 nm filter for Alexa647 labelled probes. Flow cytometry data of hybridized *P. rubra* samples were processed, analyzed and plotted in R software using the flowViz package (Sarkar et al. 2008). First, raw .fcs data files were imported and data points with a negative value for either the DAPI or A594 channel were removed (less than 1% of all the data). Then, data was transformed using the formula log(x+1) and subsequently plotted.

#### Synthetic community experiment

Synthetic community (SynCom) samples were created by mixing late exponential phase cultures of *P. mariniglutinosa, A. fischeri, V. anguillarum, E. coli* and *P. rubra* in ratios of 1:1:1:1:1 (20% *P. rubra* abundance sample) and 12:12:12:12:1 (2% *P. rubra* abundance) based on OD600 values of the cultures. After mixing, a subsample of the SynCom samples was directly fixed in a 1% paraformaldehyde solution for one hour at room temperature, washed twice with PBS and stored at -20°C in 50% EtOH-PBS until use for SecMet-FISH. The remainder of the sample was used for DNA extraction and subsequent PCR and probe synthesis.

#### Fluorescence activated cell sorting (FACS)

Cell sorting was achieved on a SONY MA900 cell sorter through a 70 µm sorting chip with sheath fluid (ClearSort^TM^, PBS) and using the semi-yield sorting mode. The cell sorter was calibrated and optically aligned using polystyrene Automatic Setup Beads (Sony) prior to each sorting experiment. The backscatter channel was used as trigger and signals were recorded using a 405 nm laser and 450/50 nm filter for DAPI signal and a 561 nm laser and 617/30 nm filter for Alexa594 signal. Before sorting, a subsample of the target sample was recorded to set a gate for sorting: first, a parent gate was drawn to distinguish noise from cells using the DAPI channel, and a secondary gate was set in the DAPI vs Alexa594 plot to select the top 10% events in the Alexa594 channel. This gate was subsequently used to sort cells from the sample. After sorting, cells were collected by centrifugation (10 000 × *g* for 2 min.) and resuspended in a total volume of 100 µL and stored at −80°C until DNA extraction. Four samples were included in the analysis: two with the non-sense probe, and two with the *P. rubra* AD/KS probes.

#### DNA extraction and 16S rRNA gene PCR on sorted populations

DNA from FACS sorted cells was extracted using the microvolume DNA extraction method using the physical lysis protocol (Bramucci et al. 2022). In brief, a cell suspension of 100 µL was lysed using a lysis buffer consisting of 0.17 M KOH and 0.013 M dithiothreitol, pH 12, for 10 min. at room temperature, followed by a freeze-thaw cycle at −80°C and 5 min. incubation at 55°C. Lysates were neutralized using 2.5 M Tris-HCl buffer, pH 5.0 and DNA was captured using Agencourt AMPure XP magnetic beads (Beckman Coulter, USA) in a 1:1.6 ratio with an extended DNA binding incubation time of 10 min.

As for biosynthetic domain PCRs, amplifications were done using HotStarTaq polymerase (Qiagen) in 50 µL reaction volumes. Detailed information on primer sequences, thermal cycling conditions, and reagent and template concentrations are summarized in Table S1. The PCR amplicons were run on a 1% agarose gel alongside a positive control (*P. rubra* gDNA) and two negative controls (H_2_0 and PBS).

#### Statistical analysis

To determine if there was a significant association between the DAPI and Alexa594 AD/KS probe signals, a χ^2^-test was conducted on the data acquired from ImageJ (587 data points). Additionally, the percentage of associated signals was calculated.

To test for significant differences between the Alexa594 signal values of *P. rubra* incubated with AD/KS probes and non-sense probes in flow cytometry, a two-sample t-test was used on the log(x+1) transformed flow cytometry data. Differences between the gating percentages of the SynCom sample groups were tested for statistical significance using a one-way analysis of variance (ANOVA) after a variance stabilizing log transformation of the data, followed by post-hoc Tukey's test for pairwise group comparisons.

## Results

**Figure 1.**
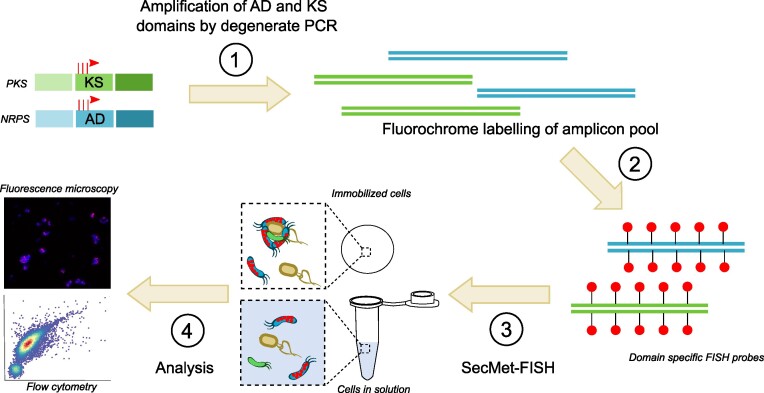
Overview of the SecMet-FISH concept for labeling, visualization, and enumeration of secondary metabolite producing microorganisms. PKS: polyketide synthase, NRPS: Non-ribosomal peptide synthetase, KS: ketosynthase domain, AD: adenylation domain.

**Figure 2.**
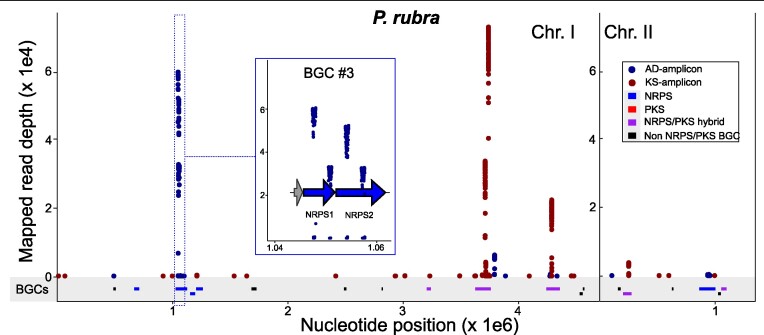
Sequence read depth of the PCR amplified adenylation (AD, blue data points) and ketosynthase (KS, red data points) domains mapped to the genome of *P. rubra* S4059. The grey panel displays the genomic position of non-ribosomal peptide synthetase (NRPS) biosynthetic gene clusters (BGCs) (blue bars), NRPS/polyketide (PKS) hybrid BGCs (purple bars) and other BGCs (black bars). The blue panel provides a higher resolution display of the mapping of AD-amplicon reads to the NRPS genes of BGC #3.

The genetic potential for production of non-ribosomal peptides and polyketides was chosen as the target feature for the development of SecMet-FISH (Fig. 1). We reasoned that the conserved and repetitive nature of the biosynthesis machinery of these compound classes would enable sufficient labelling of their genomic loci to allow for detection and quantification using fluorescence microscopy and flow cytometry. The Gram-negative marine bacterium *Pseudoalteromonas rubra* S4059 (hereafter *P. rubra*) was selected as model and target strain, as it is an excellent producer of secondary metabolites and contains multiple non-ribosomal peptide synthetase (NRPS) and polyketide synthase (PKS) BGCs in its genome (Vynne et al. 2011, Paulsen et al. 2019). To assess the applicability of using a mix of domain amplicons rather than an exact genomic locus as in direct-geneFISH, we first PCR amplified AD and KS domains from *P. rubra* using degenerate primers targeting conserved sequences of these biosynthetic domains. The lengths of the resulting amplicons were around 700 bp, as expected, and therefore similar in size to the polynucleotide probe used as proof-of-principle for direct-geneFISH (Barrero-Canosa et al. 2017). Sequence analyses of the PCR products revealed that a small fraction of the AD and half of the KS domains present in the genome of *P. rubra* (detected with antiSMASH) was successfully amplified: four out of 70 AD and five out of 10 KS domains were retrieved from the sequencing reads. The vast majority (99%) of the AD sequence reads mapped to one NRPS cluster (BGC#3, 4 mapped AD domains) and 99% of the KS sequencing reads mapped to three PKS/NRPS hybrid clusters (BGC#10, 3 mapped KS domains; BGC#11, 1 mapped KS domain and BGC#15, 1 mapped KS domain), demonstrating negligible amplification of off-targets (Fig. 2). Hence, at least nine specific loci, distributed over four BGCs, were identified as potential target hybridization sites for SecMet-FISH in *P. rubra* assuming stringent specificity.

**Figure 3.**
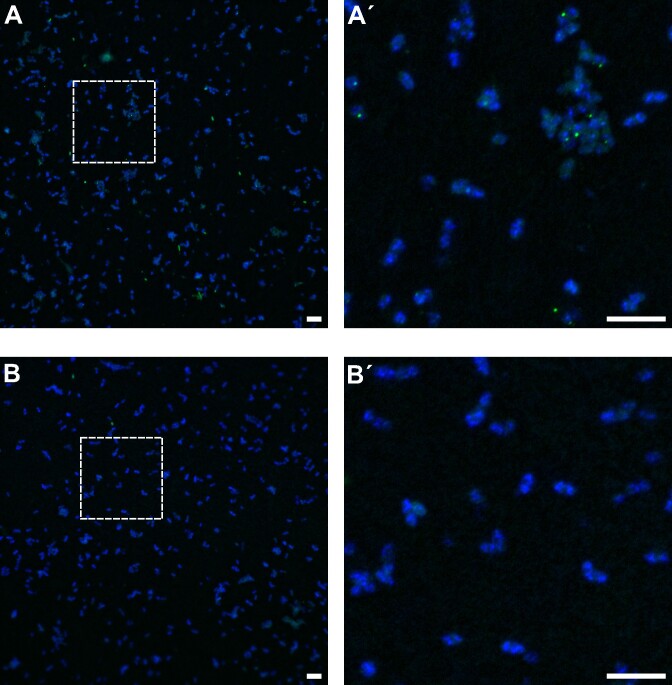
Epifluorescence microscopy of membrane filter immobilized *P. rubra* cells after hybridization with Alexa488 labelled AD/KS (A, A´) and non-sense control (B and B´) polynucleotide probes using SecMet-FISH. Cells were counterstained with DAPI DNA stain. Dashed squares in A and B are displayed in close-up in A´ and B´. Scale bar = 25 µm.

To visualize said loci with fluorescence microscopy, we generated SecMet-FISH probes by labelling the AD/KS amplicon mix with the Alexa488 dye and hybridized them in *P. rubra* cells immobilized on a membrane filter along with labelled non-sense probes as negative controls (Fig. 3). Using fluorescence microscopy, we observed the expected morphology of *P. rubra* from the DAPI-stain and observed no Alexa488 signal when adding the non-sense probes (Fig. 3B). Of much more interest, however, were the clear and discrete Alexa488 fluorescence signals observed when the amplicon probes were hybridized (Fig. 3A). These results demonstrate the ability to fluorescently label specific NRPS and PKS/NRPS hybrid BGCs, and more generally, that a mix of polynucleotide probes targeting conserved domain sequences scattered throughout the genome can successfully be used as targets for SecMet-FISH.

**Figure 4.**
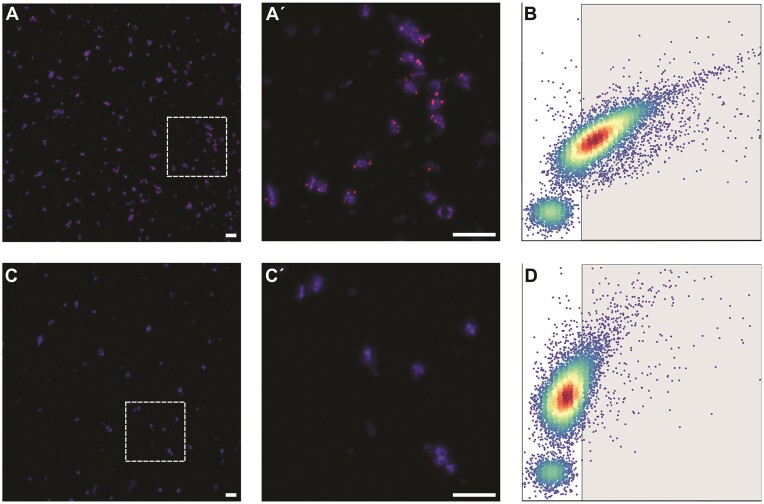
Labeling of suspended *P. rubra* cells SecMet-FISH in solution. Epifluorescence microscopy (A, A´) of *P. rubra* cells after hybridization with Alexa594 labelled AD/KS (A, A´) and non-sense control (C, C´) polynucleotide probes. Corresponding cytograms of samples hybridized with Alexa594 labelled AD/KS (B) and non-sense control (D) polynucleotide probes recorded with flow cytometry. Cells were counterstained with DAPI DNA stain. Dashed squares in A and C are displayed in close-up in A´ and C´. Scale bar = 25 µm.

Next, we explored the compatibility of SecMet-FISH with suspended cells in solution, instead of immobilized cells on a surface, with the aim of combining this method with flow cytometry. Protocols for in-solution FISH and direct geneFISH were combined (see Materials and Methods) using paraformaldehyde-fixed *P. rubra* cells. A fraction of the population showed weak, dot-like fluorescent signals (Fig. S1). In an attempt to obtain stronger signals, the protocol was optimized, and additional fluorophores (Alexa594 and Alexa647) were tested. We found that a decreased DNA denaturing temperature (80°C), a longer hybridization time (16 h) and an increased probe concentration (250 pg/µL) resulted in increased signal intensities as observed by microscopy (Fig. 4A and Fig. S1). Importantly, SecMet-FISH signals were absent when the probes were applied to *E. coli* cells analogous to their lack of AD and KS domains (Fig. S2). To further assess if SecMet-FISH allows for robust quantification of cells based their genetic potential to produce secondary metabolites, i.e. PKs and NRPs, we analyzed the degree of association between DAPI signal and AD/KS probe signal on triplicate *P. rubra* samples (Fig. S3). The distribution of 587 data points (signals from DAPI and Alexa594 channels) showed that on rare occasions, probe signal was observed dissociated from DAPI signal (2.9% of Alexa594 signals), which likely reflects hybridization to extracellular DNA. Moreover 450 out of 573 DAPI signals were associated with at least one Alexa594 signal, suggesting a labelling efficiency of 79% and a significant association between the two signals (χ^2^, *P*<0.001).

Furthermore, cell populations hybridized with the AD/KS probes were distinguishable from populations hybridized with the non-sense control probes by flow cytometry (t-test, *P* < 0.001; Fig. 4B, D), corroborating that the approach enabled specific binding to target BGCs in solution. Fluorophore-specific gates, designed such that only 1% of the cell population in the control sample would be captured in the gate, resulted in positive labelling percentages of 18, 47 and 92 for the Alexa 647, 488 and 594-labelled probes, respectively.

**Figure 5.**
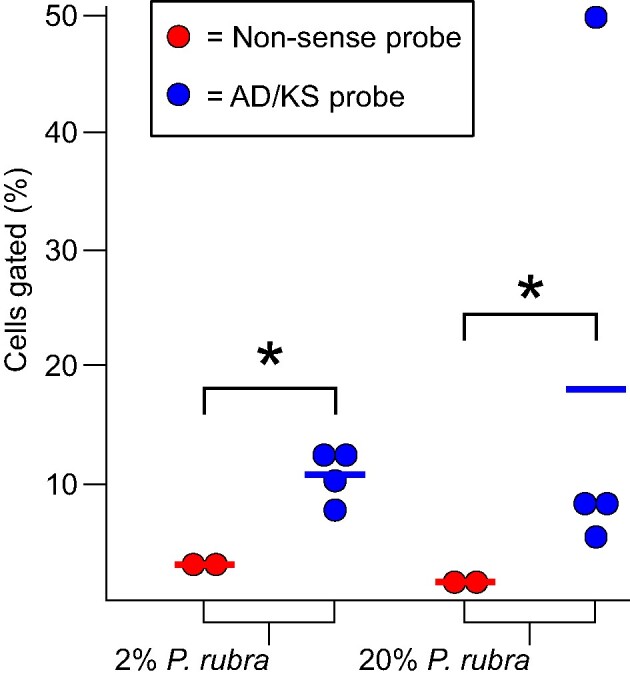
Flow cytometry derived gating percentages of SynCom samples with 2% and 20% *P. rubra* abundance after labeling with AD/KS (blue) and non-sense negative control (red) polynucleotide probes using SecMet-FISH in solution. Gates were drawn such that approximately the top 10% events from the AD/KS probe sample would be captured in the gate. Dots are replicates and horizontal bars represent the mean value. Asterisks indicate statistically significant differences (p < 0.05) between group mean values as determined by ANOVA and post-hoc Tukey's test.

In order to assess if SecMet-FISH could enable identification, enumeration, and separation of target cells from a mixed population, we created two distinct SynCom samples, each containing five bacterial strains including *P. rubra* at abundances of 2% and 20%, respectively. SynCom subsamples were used as starting material for DNA extraction, PCR amplification of AD and KS domains and probe synthesis using the Alexa594 fluorophore. Subsequently, SynCom samples were subjected to SecMet-FISH in solution, and hybridized subsamples were analyzed by flow cytometry. Flow cytometry analysis revealed that hybridization with the AD/KS probes resulted in larger percentages of cells captured in the high Alexa594 signal gate compared to hybridization with the negative control probes (Tukey's test, 2% *P. rubra* SynCom: *P* = 0.043; 20% *P. rubra* Syncom: p = 0.011; Fig. 5), which indicates that the targeted labelling of AD/KS domains in the genomes allow for the successful discrimination between community members based on BGC content. We did, however, not observe a clear difference in positively gated cell fractions between the 20% and 2% *P. rubra* SynCom samples labelled with AD/KS probes (Tukey's test, *P* = 0.97).

To determine if DNA from labelled, and hence paraformaldehyde treated, and sorted cells where of sufficient quality for downstream analysis, we sorted cells out using FACS, extracted DNA and performed 16S rRNA gene PCRs. We observed strong bands for all four samples, suggesting that paraformaldehyde fixation does not crosslink DNA to a degree that prohibits downstream analyses of SecMet-FISH sorted cells (Fig. S4).

**Figure 6.**
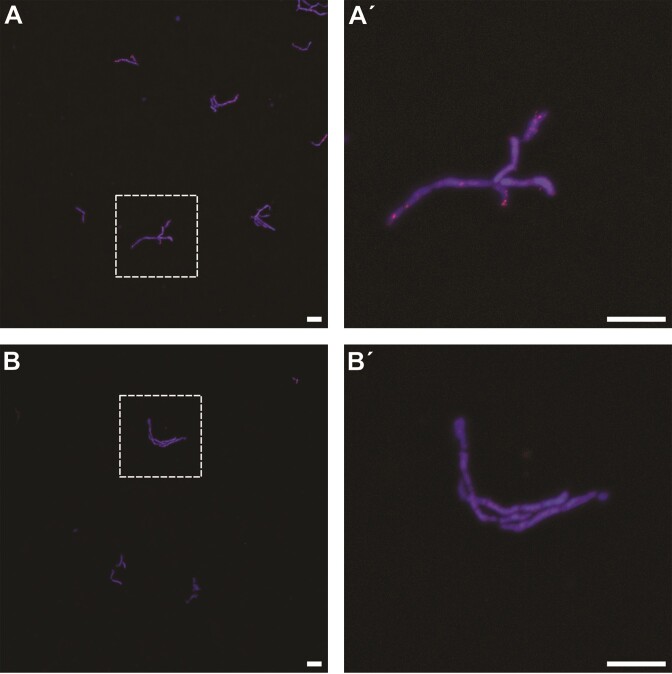
Epifluorescence microscopy of suspended *S. coelicolor* cells after hybridization with Alexa594 labelled AD/KS (A, A´) and non-sense control (B and B´) polynucleotide probes using in-solution SecMet-FISH. Cells were counterstained with DAPI DNA stain. Dashed squares in A and B are displayed in close-up in A´ and B´. Scale bar = 25 µm.

FISH protocols can vary considerably based on the target bacterial cells, most notably due to differences in the cell wall structure between Gram-positive and Gram-negative bacteria. Towards a broader application potential of SecMet-FISH, we applied and optimized our protocol targeting Gram-positive bacteria, for which we used the secondary metabolite producing model strain *Streptomyces coelicolor* A3. Adaptations for hybridization targeting *S. coelicolor* were inclusion of a lysozyme treatment for cell wall permeabilization, adjustment of the denaturation temperature to 75°C and adjustment of the formamide concentration in the hybridization buffer to 50% (v/v). Epifluorescence microscopy analysis of *S. coelicolor* cells hybridized with the specific AD/KS probe mix revealed multiple SecMet-FISH signals dispersed over the filamentous cells (Fig. 6). Similar looking signals were not observed for cells hybridized with the non-sense control probe, demonstrating that the SecMet-FISH method was successfully adapted for specific labelling of Gram-positive bacteria as well.

## Discussion

The development of catalyzed reporter deposition (CARD)-geneFISH (Moraru et al. 2010) and more recently direct-geneFISH (Barrero-Canosa et al. 2017) has opened up the possibility for detection and localization of microbial cells with specific functional gene profiles in environmental samples, as well as the coupling of this potential to specific microbial taxa. One limitation in the applicability of this method for high-throughput detection and quantification, e.g. using flow cytometry, is that hybridization of labelled probes to their target sequences occur inside immobilized cells rendering them out of reach for downstream analyses beyond direct imaging. In addition, the limited signal from single-copy target sequences poses a significant challenge for the utility of contemporary geneFISH approaches with flow cytometry or FACS. In this work, we capitalized on the developments within in-solution Flow-FISH approaches (Yilmaz et al. 2010, Haroon et al. 2013, Freen-van-Heeren 2021) as well as the conserved and repetitive nature of NRPS and PKS BGCs to increase the targeted loci, enabling detection and quantification of cells labelled based on their biosynthetic gene content using both fluorescence microscopy and flow cytometry. Combining SecMet-FISH with optical trapping or FACS may thus allow for e.g. targeted metagenomics or single-cell genomics on microbial community members rich in BGCs, as well as retrieval of genetic material for subsequent cloning and expression (Grieb et al. 2020) in the future. Moreover, SecMet-FISH is applicable with immobilized cells and thus allows for the generation of data describing the spatial distribution of secondary metabolite producers in complex microbial communities.

As a proof-of-concept, we used the biosynthetic capabilities of *P. rubra* as a target functional genetic trait. Specifically, we successfully labelled at least four BGCs encoding the production of non-ribosomal peptide and polyketide/non-ribosomal peptide hybrid secondary metabolites, through the conserved AD and KS domains within the BGCs. Despite the use of degenerate primer pairs that have been used extensively for profiling of the biosynthetic potential of various environmental niches (Piel 2002, Ayuso-Sacido and Genilloud 2005, Charlop-Powers et al. 2014, 2016, Lemetre et al. 2017, Bech et al. 2020, Geers et al. 2022, 2023), sequence analysis of the amplicons showed that only about 5% and 50% of AD and KS domains, respectively, were amplified from *P. rubra*. In accordance with these findings, it has been shown before, for example through *in silico* PCR, that degenerate primer sets targeting AD and KS domains often amplify less than 40% of the targets (Geers et al. 2022). Under the conservative assumption that the generated probes only bind to the specific target sequence (specificity of 100%), our approach is unable to label and capture the full diversity of AD and KS domains, and the cells harboring them. This also implies that community members harboring more BGCs than the nine targets amplified here, e.g. uncultured Acidobacteriota and Verrucomicrobiota (Crits-Christoph et al. 2018, 2022, Waschulin et al. 2022), could produce a stronger signal than obtained in the present proof-of-concept setup. Importantly, the nine genetic target loci amplified from the genome of *P. rubra* proved to be sufficient for signal detection by epifluorescence microscopy and flow cytometry, although the labelling efficiency was only 79% according to the signal association analysis.

A major distinction between conventional direct-geneFISH and SecMet-FISH is that washing steps in-solution necessitate repetitive centrifugation and resuspension steps. We speculate that this, in combination with the high temperature incubations during denaturation of the target DNA is sufficient to make the Gram-negative *P. rubra* cells permeable to the polynucleotide probes. Indeed, we observed cell lysis when cells were treated with lysozyme and/or incubated at temperatures above 80°C. Reports on bacterial cell wall damage due to centrifugation support this hypothesis (Peterson et al. 2012). Optimizations of the protocol included an increase in probe concentration and hybridization time. The necessity for these adaptations might be explained by the effective probe concentration: assuming equal amplification of ***n*** unique and equally distributed AD and KS domains, the expected value of each unique probe will be 1/***n***, which here implies that the effective probe concentration decreases as the complexity of the system increases. Additionally, the abundance of each domain is likely to follow the same exponential laws governing the abundances of their hosts, resulting in extreme skewness of domains as well. As hybridization kinetics are dependent on (effective) probe concentration (Wetmur and Fresco 1991), increased hybridization time and probe concentrations are needed to compensate for a decreased effective probe concentration. We observed the best signal detection using the Alexa594 fluorophore in the proof-of-concept experiments, while Alexa647 labelled probes resulted in poor signal detection. This corroborates previous observations (Barrero-Canosa et al. 2017), and highlights the importance of fluorophore sensitivity in polynucleotide probe hybridizations.

In our SynCom experiment we attempted to use SecMet-FISH to label and differentiate the target strain *P. rubra* from a mixed sample containing five bacterial strains. Flow cytometry analysis showed significant differentiation between labelled and non-labelled cells, showing that SecMet-FISH in combination with flow cytometry is an efficient way of differentiating bacterial cells based on their biosynthetic gene repertoire. Hence, the complementation of SecMet-FISH with FACS could facilitate selective extraction and enrichment of bacteria from a complex microbial community based on their genetic potential for secondary metabolite production. With this aim, a different fluorescent labelling strategy has recently been proposed for the enrichment of secondary metabolite producers from tunicate and nudibranch microbiomes (Kim et al. 2021, Džunková et al. 2023). In this approach, the carrier protein involved in non-ribosomal peptide and polyketide biosynthesis was directly labelled using a fluorescent analog molecule as probe (Kim et al. 2021). The main difference between this approach and SecMet-FISH is the requirement for expression of the biosynthetic pathway in order to achieve fluorescent labelling of the target cells. Although labelling of functional biosynthetic enzymes has the advantage of gaining ecological insights into the *in situ* production of secondary metabolites, the approach is likely to miss a substantial fraction of community members with a broad secondary metabolite repertoire, as secondary metabolism is a tightly regulated process (Van Wezel and McDowall 2011, Santamaria et al. 2022).

## Conclusion

We report the development of SecMet-FISH, a method that enables the detection and distinct enumeration of bacteria carrying the genetic capacity to synthesize polyketide and non-ribosomal peptide metabolites. By (i) labelling multiple loci at once through synthesis of polynucleotide probes after degenerate PCR amplification of conserved sequences and (ii) hybridization within cells that are suspended in solution, SecMet-FISH offers the possibility for high-throughput discrimination and analysis of secondary metabolite producers by flow cytometry of labelled cells. SecMet-FISH can be adjusted to efficiently label BGCs within Gram-negative and Gram-positive cells. Moreover, the approach may be further extended to hybridization of other functional domains or genes or it may be combined with fluorescence assisted trapping or sorting for the specific acquisition of genetic material from secondary metabolite producers from environmental samples in the future.

## Supplementary Material

fiae038_Supplemental_File

## Data Availability

Sequencing data are available in the Sequencing Read Archive (SRA) under accession number PRJNA996863.
