# Supplementary material for: SecMet-FISH: labeling, visualization, and enumeration of secondary metabolite producing microorganisms
Source: FEMS Microbiol Ecol. 2024 Mar 15;100(5):fiae038. doi: 10.1093/femsec/fiae038 (PMC11004939; doi:10.1093/femsec/fiae038)
Supplement: fiae038_Supplemental_File [file fiae038_supplemental_file.pdf]

Supporting information for

SecMet-FISH: Labeling, visualization, and enumeration of secondary metabolite producing microorganisms through fluorescence in situ hybridization

Yannick Buijs, Aileen Ute Geers, Iuliana Nita, Mikael Lenz Strube, Mikkel Bentzon-Tilia

Table S1. PCR reagent concentrations, thermocycling conditions and primer sequences.

Reagent	Volume		Temperature	Time	
H_2O	Up to 50 µL		95 °C	15:00 min	
HotStarTaq 2x	25 µL		94 °C	0:30 min	Variable number of cycles[1]
Forward primer (10 µM)	Variable[1]		Variable	0:40 min	
Reverse primer (10 µM)	Variable[1]		72 °C	0:30 min	
BSA (20 mg/mL)	2 µL		72 °C	5:00 min	
DNA template	Variable[2]				

[1]: for the details specific to each PCR reaction, see table below
[2]: for the details specific to each PCR reaction, see the materials and methods section in the main text

	V3V4	AD	KS
Forward primer sequence	CCTACGGGNGGCWGCAG	GCSTACSYSATSTACACSTCSGG	MGNGARGCNNWNSMNATGGAYCCNCARCANMG
Reverse primer sequence	GACTACHVGGGTATCTAATCC	SASGTCVCCSGTSCGGTA	GGRTCNCCNARNSWNGTNCCNGTNCCRTG
Primer reference	(Herlemann et al., 2011)	(Ayuso-Sacido & Genilloud, 2005)	(Piel, 2002)
Primer concentration	0.32 µM	0.64 µM	1.28 µM
Annealing temperature	56 °C	55 °C	54 °C
Template concentration pure strains + syncom samples	0.4 ng/µL	0.4 ng/µL	0.8 ng/µL
Template volume µ-volume DNA extracts	4	-	-

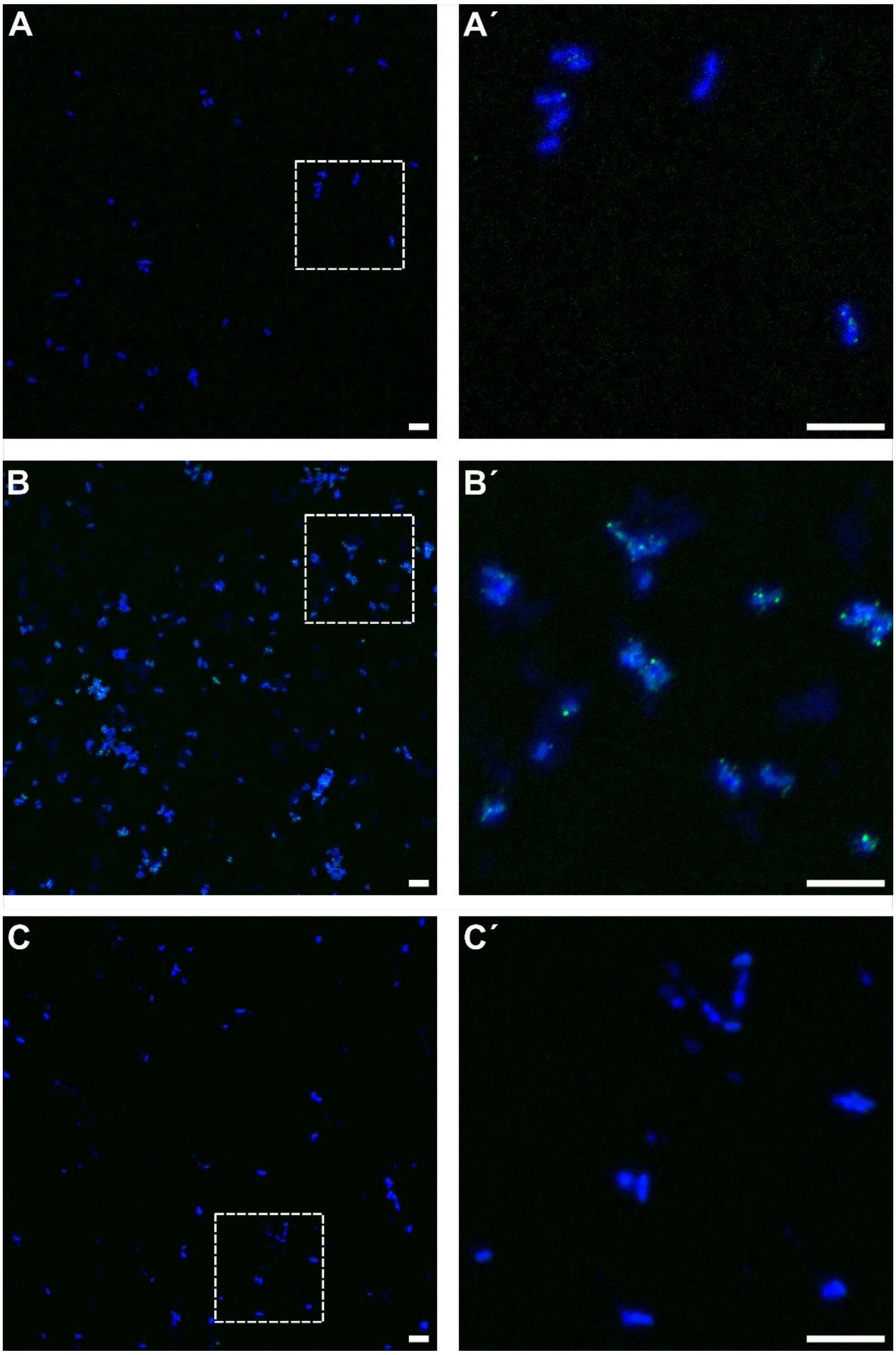

Figure S1. Epifluorescence microscopy of suspended *P. rubra* cells after hybridization with Alexa488 labeled AD/KS polynucleotide probes before protocol optimization (**A, A´**), after protocol optimization (**B, B´**) and after hybridization with Alexa488 labeled non-sense negative control polynucleotide probes (**C, C´**) using SecMet-FISH. Cells were counterstained with DAPI DNA stain. Dashed squares in A and B are displayed in close-up in A´ and B´. Scale bar = 25 µm.

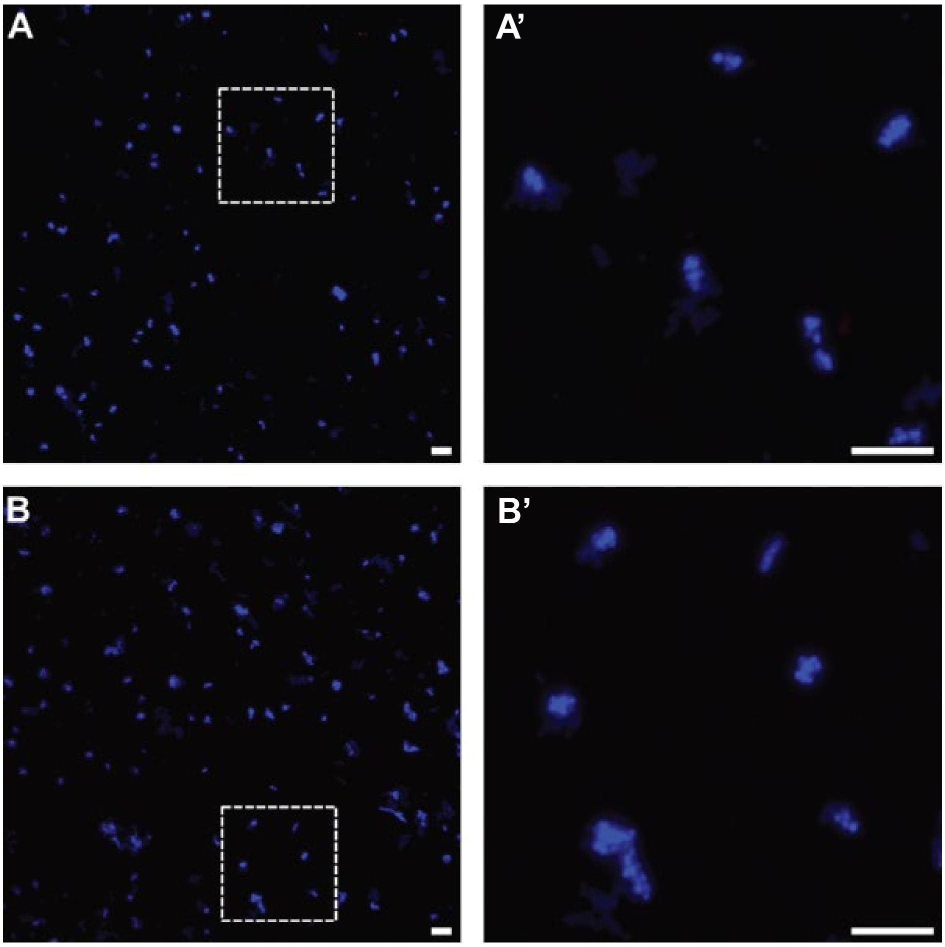

Figure S2. Epifluorescence microscopy of suspended *E. coli* cells after hybridization with Alexa594 labeled AD/KS (*P. rubra* specific) (A, A´) and non-sense negative control polynucleotide probes (B, B´) using in-solution SecMet-FISH. Cells were counterstained with DAPI DNA stain. Dashed squares in A and B are displayed in close-up in A´ and B´. Scale bar = 25 µm.

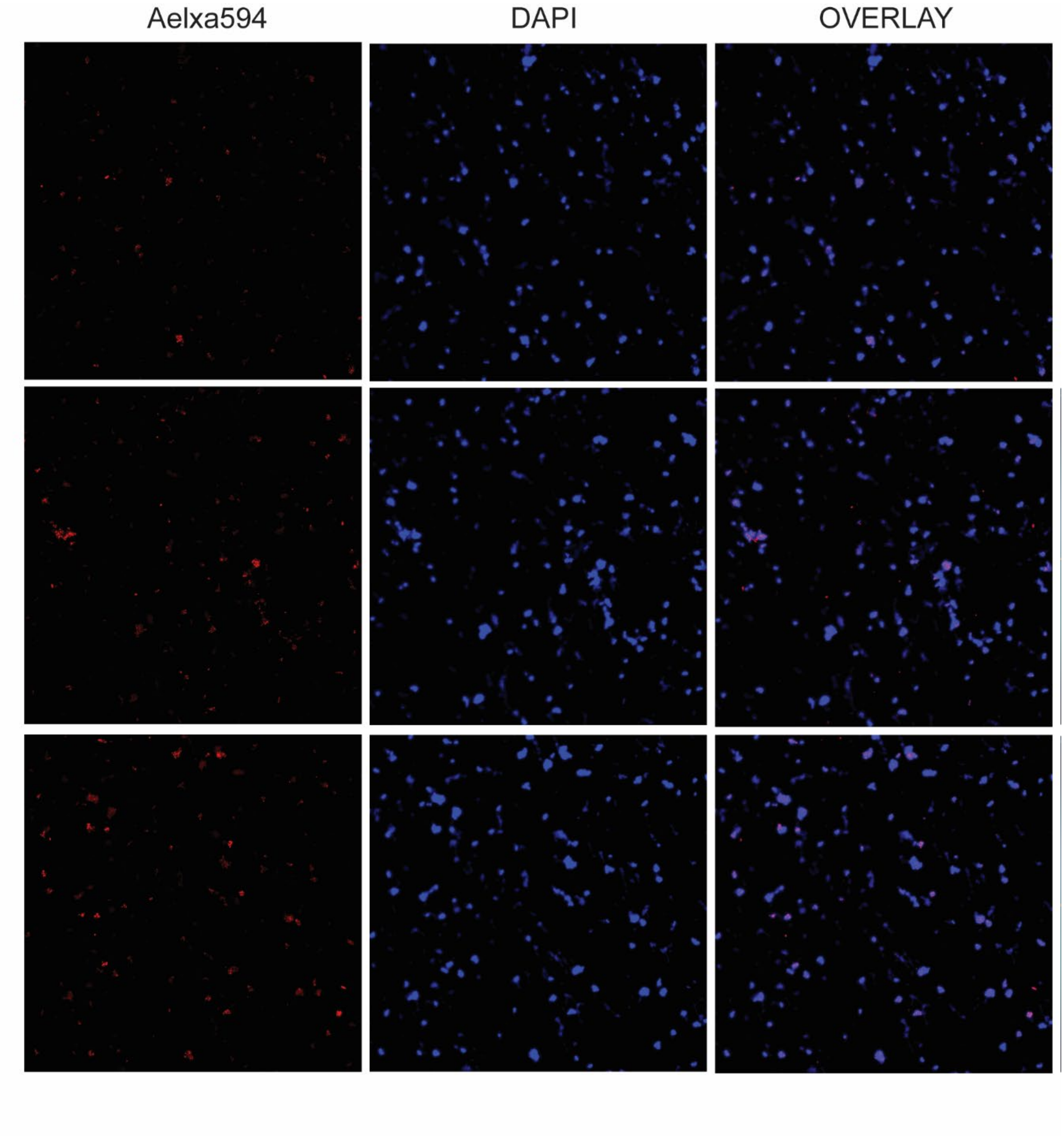

Figure S3. Epifluorescence microscopy of suspended *P.rubra* cells after hybridization with Alexa594 labeled AD/KS probes and DAPI. 2.9% of the detected Alexa594 signals occurred dissociated from DAPI signal, whereas 79% occurred in association with the DAPI signal.

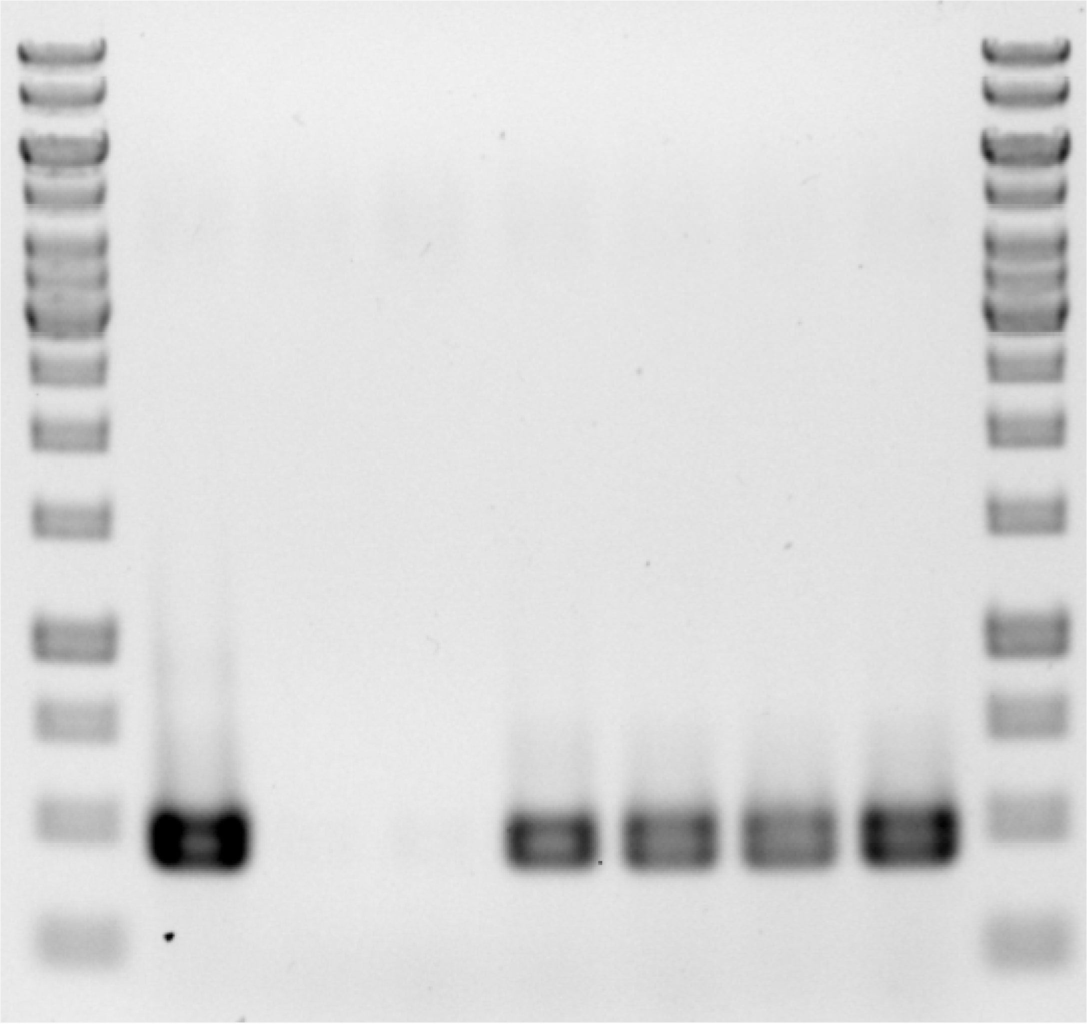

Figure S4. V3V4 16S rRNA gene PCR on DNA from labelled and sorted cells. Wells from left to right: 1: ladder, 2: Positvie control (*P. rubra* gDNA), 3: negative control 1(H_20), 4: negative control 2 (PBS), 5+6: SecMet-FISH sorted cells with Non-sense probe, 7+8: SecMet-FISH sorted cells with specific probe, 9: ladder.
